# Supplementary material for: Cell-autonomous and non-cell-autonomous effects of Arginase 2 on cardiac aging
Source: eLife. 2025 Nov 4;13:RP94794. doi: 10.7554/eLife.94794 (PMC12585178; doi:10.7554/eLife.94794)
Supplement: Supplementary file 1. [file elife-94794-supp1.docx]

**Supplementary File 1. Baseline comparison between wt and *Arg2^-/-^* mice under Langendorff recordings**

| **N = 5**  **Each group** | **Pmax (mm Hg)** | **Pmin (mm Hg)** | **DP**  **(ms)** | **SEP**  **(ms)** | **DFP (ms)** | **CT**  **(ms)** | **RT**  **(ms)** | **dPmax** | **dPmin** | **CI** | **HR**  **(beats/min)** |
| --- | --- | --- | --- | --- | --- | --- | --- | --- | --- | --- | --- |
| **wtO F** | 71.8±22.8 | 9.8±  2.7 | 65.8±19.7 | 43.6±  15.5 | 227±  115 | 28.4±2.15 | 68.2±  3.9 | 1807±  854 | -1133±  566 | 63±  5.4 | 214.8±  55.6 |
| ***Arg2^-/-^*O F** | 91.2±20.6 | 7.4±5.2 | 85.0±23.3 | 41.8±  6.8 | 270±  135 | 31.8±1.2 | 67.4±  6.7 | 2340±  707 | -1759  ±569 | 57.6±2.6 | 189.6±  47.6 |

* DP = developed pressure

* SEP = systolic ejection period (ms)

* DFP = diastolic filling time (ms)

* CT = contraction time (ms)

* RT = relaxation time (ms)

* CI = contractility Index

* HR = Heart rate
