# Supplementary material for: Cell-autonomous and non-cell-autonomous effects of Arginase 2 on cardiac aging
Source: eLife. 2025 Nov 4;13:RP94794. doi: 10.7554/eLife.94794 (PMC12585178; doi:10.7554/eLife.94794)
Supplement: Supplementary file 2. [file elife-94794-supp2.docx]

**Supplementary File 2. The RT-PCR primer sequences**

| **The following primer sequences of mouse (m) and human (h) origin were used:** | | |
| --- | --- | --- |
| **Gene** | **Forward primer sequences (5’-3’)** | **Reverse primer sequences (5’-3’)** |
| *m-Arg1* | 5′-GGA ATC TGC ATG GGC AAC CTG TGT-3′ | 5′-AGG GTC TAC GTC TCG CAA GCC A-3′ |
| *m-Arg2* | 5′-CCC CTT TCT CTC GGG GAC AGA A-3′ | 5′-GAA AGG AAA GTG GCT GTC CA-3′ |
| *m-Adgre1* | 5′-TGG CTG CCT CCC TGA CTT TC-3′ | 5′-CAA GAT CCC TGC CCT GCA CT-3′ |
| *m-Il1b* | 5′-GCA ACT GTT CCT GAA CTC AAC T-3′ | 5′-TCT TTT GGG GTC CGT CAA CT-3′ |
| *m-Mcp1* | 5′-AGC ACC AGC CAA CTC TCA C-3′ | 5′-TCT GGA CCC ATT CCT TCT TG-3′ |
| *m-Tnfa* | 5′-GGC AGG TCT ACT TTG GAG TCA TTG C-3′ | 5′-ACA TTC GAG GCT CCA GTG AAT TCG G-3′ |
| *m-Tgfb1* | 5′-TGG AGC AAC ATG TGG AAC TC-3′ | 5′-CAG CAG CCG GTT ACC AAG-3′ |
| *m-Col1a* | 5′-TGGCCAAGAAGACATCCCTGAAGTC-3′ | 5′-GGCAGATACAGATCAAGCATACCTCGG-3′ |
| *m-Col3a* | 5′-CAAACACGCAAGGCAATGAGACTACC-3′ | 5′-AGGGCCAATGTCCACACCAAATTC-3′ |
| *m-Col4* | 5′-CAGATTCCGCAGTGCCCTA-3′ | 5′-GGAATAGCCGATCCACAGTGAG-3′ |
| *m-Fib* | 5′-ACCTCTGCAGACCTACCCAG-3′ | 5′-TTGGTGATGTGTGAAGGCTC-3′ |
| *m-Mmp2* | 5′-ACCTGAACACTTTCTATGGCTG-3′ | 5′-CTTCCGCATGGTCTCGATG-3′ |
| *m-Mmp9* | 5′-GCAGAGGCATACTTGTACCG-3′ | 5′-TGATGTTATGATGGTCCCACTTG-3′ |
| *m-Rps12* | 5′-GAA GCT GCC AAA GCC TTA GA-3′ | 5′-AAC TGC AAC CAA CCA CCT TC-3′ |
| *m-Gapdh* | 5′-ACCCAGAAGACTGTGGATGG-3′ | 5′-ACACATTGGGGGTAGGAACA-3′ |
| *h-ARG2* | 5′-GGCTGAGGTGGTTAGCAGAG-3′ | 5′-CTGGCTGTCCATGGAGATTT-3′ |
| *h-COL1a* | 5′-ACCTCCTGGCAAAGATGGAA-3′ | 5′-GAGCCCTCAGATCCTCTTTCAC-3′ |
| *h-COL3a* | 5′-GTTCGTGACCGTGACCTCG-3′ | 5′-TCTTGTCCTTGGGGTTCTTGC-3′ |
| *h-COL4* | 5′-CAATATGAAAACCGTAAAGTGCCTTATA-3′ | 5′-CAGCAAGTAGAGGTCAATGAAGCA-3′ |
| *h-GAPDH* | 5′-GGTCTCCTCTGACTTCAACA-3′ | 5′-AGCCAAATTCGTTGTCATAC-3′ |
