# Supplementary material for: Cell-autonomous and non-cell-autonomous effects of Arginase 2 on cardiac aging
Source: eLife. 2025 Nov 4;13:RP94794. doi: 10.7554/eLife.94794 (PMC12585178; doi:10.7554/eLife.94794)
Supplement: Supplementary file 3. [file elife-94794-supp3.docx]

**Supplementary File3. Antibody dilutions used for immunoblotting and immunofluorescence staining**

| **Antibody target** | **Dilution** |
| --- | --- |
| ARG2 (#55003, cell signaling) | WB 1:1000;  IF 1:100 |
| IL-1β (ab9722, Abcam) | WB 1:1000  IF 1:100 |
| HIF-1α (#36169, cell signaling) | WB 1:1000 |
| a-SMA (ab7817, Abcam) | IF 1:200 |
| α-Tubulin (T5168, Sigma) | WB 1:4000 |
| Cardiac Troponin T (ab8295, Abcam) | IF 1:100 |
| MAC-2 (14-5301-82, Invitrogen) | IF 1:200 |
| CD31 (sc-1506, Santa Cruz) | WB 1:1000;  IF 1:100 |
| P16 (sc-81156, Santa Cruz) | IF 1:50 |
| LYVE1 (14-0443-82; ThermoFisher) | IF 1:100 |
| CCR2 (ab223050, Abcam) | IF 1:100 |
| F4-80 (30325S, cell signaling) | IF 1:100 |
| CD68 (MA5-13324KP1; ThermoFisher) | WB 1:100 |
| PDGF-Rα (AF1062, R D systems) | IF 1:200 |
| Vimentin (ab8978, Abcam) | WB 1:1000;  IF 1:100 |
| Vinculin (MCA465GA, Bio-Rad) | WB 1:5000 |
| VE-Cadherin (ab33168, Abcam) | WB 1:1000 |
| N-Cadherin (#13116, cell signaling) | WB 1:1000 |
| GAPDH (10R-G109A; Fitzgerald Biosciences) | WB 1:100,000 |
| SNAIL + SLUG (ab180714, Abcam) | WB 1:500 |
| IRDye 800-conjugated affinity purified goat anti-rabbit IgG  (9263221, BioConcept) | WB 1:5,000 |
| Alexa fluor 680-conjugated goat anti-mouse IgG (A-21057, Invitrogen) | WB 1:5,000 |
| Alexa Fluor 488-conjugated goat anti-rabbit IgG (H+L) secondary Ab (A-11008, Thermo Fisher Scientific) | IF 1:400 |
| Alexa Fluor 594-conjugated goat anti-rabbit IgG (H+L) secondary Ab (A-11012, Thermo Fisher Scientific) | IF 1:400 |
| Alexa Fluor 488-conjugated goat anti-mouse IgG (H+L) secondary Ab (A-11001, Thermo Fisher Scientific) | IF 1:400 |
| Alexa Fluor 568-conjugated goat anti-mouse IgG (H+L) secondary Ab (A-11031, Thermo Fisher Scientific) | IF 1:400 |
| Alexa Fluor 488-conjugated donkey anti-goat IgG (H+L) secondary Ab (A11055, Thermo Fisher Scientific) | IF 1:400 |
