# Supplementary material for: Cell-autonomous and non-cell-autonomous effects of Arginase 2 on cardiac aging
Source: eLife. 2025 Nov 4;13:RP94794. doi: 10.7554/eLife.94794 (PMC12585178; doi:10.7554/eLife.94794)
Supplement: Supplementary file 6. [file elife-94794-supp6.pdf]

Analysis Report for Cell Line Authentication

1. Sponsor

Mr. Duilio Michele Potenza
Université de Fribourg
Groupe Yang
Ch. du Musée 5
1700 Fribourg

2. Analysis Report

Report ID: 02874_018003
Report Version: 01
Issue Date: 08.07.2025
Report approved by: Agatha Borejdo

3. Descriptions

Customer Test Item ID: THP1-KO
Analysis Method: Profiling of the human cell lines was done using highly polymorphic short tandem repeat loci (STRs). STR loci were amplified using the PowerPlex® 16 HS System (Promega). Fragment analysis was done on an ABI3730xl (Life Technologies) and the resulting data were analyzed with GeneMarker HID software (Softgenetics).

4. Analysis Results

4.1. Summary Table of the STR Profile

Locus	*Chromosomal Location*	*Core STR Marker*	*Customer Sample Typed Alleles*	*Database Alleles*	*Comments*
D3S1358	Chr03		15/17	15/17	
TH01	Chr11	Yes	8/9.3	8/9.3	
D21S11	Chr21		30/31.2	30/31.2	
D18S51	Chr18		13/14	13/14	
Penta_E	Chr15		11/15	11/15	
D5S818	Chr05	Yes	11/12	11/12	
D13S317	Chr13	Yes	13	13	
D7S820	Chr07	Yes	10	10	
D16S539	Chr16	Yes	11/12	11/12	
CSF1PO	Chr05	Yes	11/13	11/13	
Penta_D	Chr21		10/12	10/12	
AMEL	X/Y	Yes	X/Y	X/Y	
vWA	Chr12	Yes	16	16	
D8S1179	Chr08		10/14	10/14	
TPOX	Chr2	Yes	8/11	8/11	
FGA	Chr04		24/25	24/25	

Microsynth AG
Schützenstrasse 15 · CH-9436 Balgach · Phone +41 71 722 83 33 · info@microsynth.ch · www.microsynth.com

4.2. Electropherogram

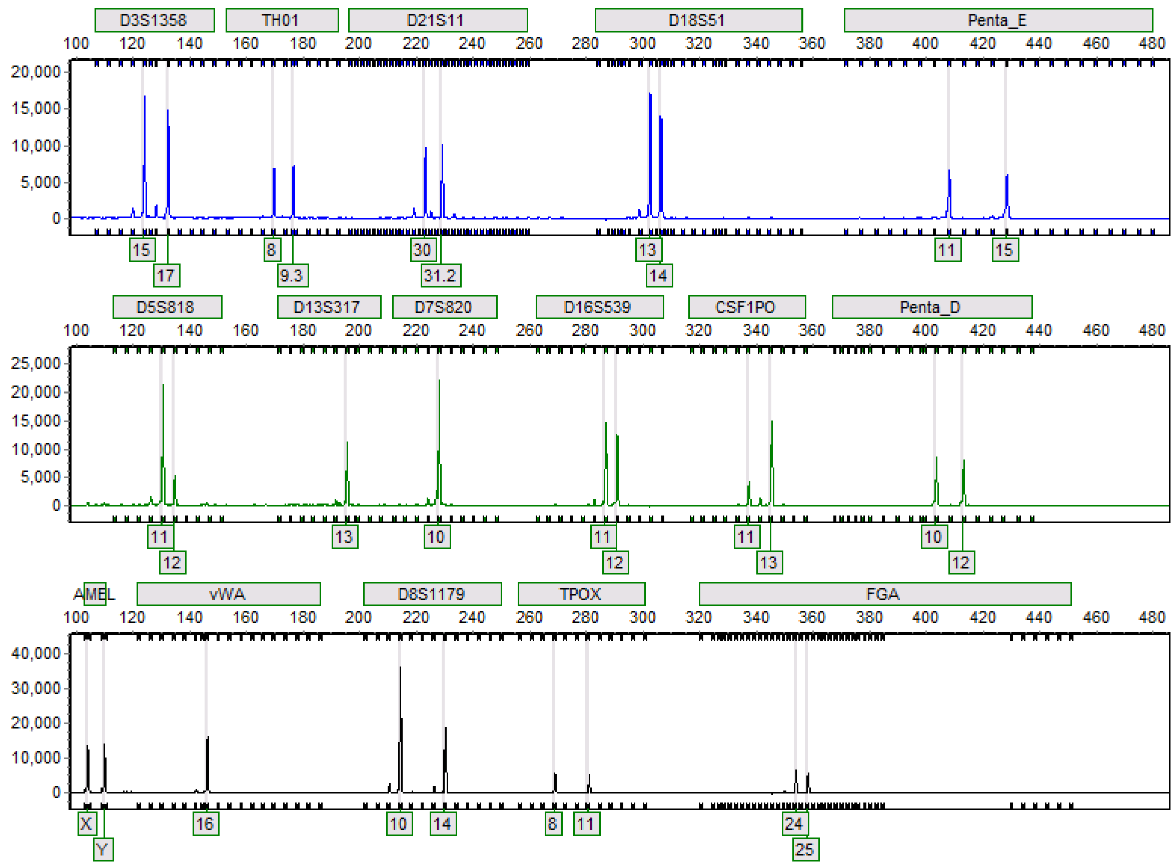

5. Conclusion

According to our analysis of the submitted sample there is no detectable contamination with human origin.

The analyzed data of the submitted sample match 100 % to the DNA profile of the cell line THP-1 (Cellosaurus, RRID:CVCL_0006_[ATCC]).

6. Customer Comment

No specific customer comments were provided for this test item

7. Glossary

Short Tandem Repeats (STRs)

Short tandem repeats (STRs) consist of a DNA motif of 2-13 bases that are repeated up to several hundred times. The number of repeats in a STR is highly variable among individuals, resulting in fragment length differences if amplified using PCR. These differences in fragment lengths at different loci are used for profiling the cell lines.

Stutter Peaks

Stutter peaks are small peaks which occur immediately before or after the true peak. Stutter peaks are commonly caused by a slippage of the polymerase during the PCR amplification.

Detection of Cell Line Mixtures

Contamination of one cell line by one or several other cell lines can be detected down to a frequency of the contaminating cell line of 10%. Typically, cell line mixtures will result in STR profiles including three or more peaks for single or multiple loci. If Microsynth notices a possible contamination of a cell line, we will comment the finding in the conclusion part of the analysis.

Peak height ratio

Peak height ratio <25 % (to the highest peak within a STR) is mentioned in the summary table (comments). Peak height ratios <25% need not necessarily have an effect on the behaviour or characteristics of the cell line. A small peak height may be due to reduced amplification efficiency, for example resulting from a mutation in the primer site. The reason for the difference in peak heights observed, however, would need some in depth analysis of the test item.

8. General Comment

The results refer only to the portion of the sample Microsynth has analyzed. The analysis results might not be assigned unconditionally to the whole sample. Microsynth shall not in any event be liable for incidental, consequential or special damages in relation to carried out analyses and corresponding results.

This report is the confidential property of the client addressed. The report may only be reproduced in full. Publication of extracts from this report is not permitted without written approval from Microsynth.

9. Compliance and Quality Assurance Statement

All aspects of this study were in accordance with ISO 9001:2015 standards. All the applied equipment is qualified and calibrated. The applied methods are validated.
