# Supplementary material for: Cell-autonomous and non-cell-autonomous effects of Arginase 2 on cardiac aging
Source: eLife. 2025 Nov 4;13:RP94794. doi: 10.7554/eLife.94794 (PMC12585178; doi:10.7554/eLife.94794)
Supplement: Supplementary file 8. [file elife-94794-supp8.pdf]

Analysis Report for Cell Line Authentication

1. Sponsor

Mr. Duilio Michele Potenza
Université de Fribourg
Groupe Yang
Ch. du Musée 5
1700 Fribourg

2. Analysis Report

Report ID: 02874_018005
Report Version: 01
Issue Date: 08.07.2025
Report approved by: Agatha Borejdo

3. Descriptions

Customer Test Item ID: BMDM_KO
Analysis Method: Profiling of the mouse cell line was done using highly polymorphic short tandem repeat loci (STRs) published in Almeida et al. (2019). Fragment analysis was done on an ABI3730xl (Life Technologies) and the resulting data were analyzed with GeneMarker software (Softgenetics).

4. Analysis Results

4.1. Summary Table of the STR Profile

Locus	*Chromosomal Location*	*Customer Sample Typed Fragment Lengths*	*Database Alleles*	*Comments*
M_18-3	Chr18	16	16	
M_4-2	Chr04	20.3	20.3	
M_6-7	Chr06	15	15	
M_3-2	Chr03	14	14	
M_19-2	Chr19	13	13	
M_7-1	Chr07	26.2	26.2/27.2	
M_1-1	Chr01	16	16	
M_Sex	SRY	X	N/A	
M_8-1	Chr08	16	16	
M_2-1	Chr02	15/16	16	
M_15-3	Chr15	22.3	22.3	
M_6-4	Chr06	17/18	18/19	
M_11-2	Chr11	16/17	16	
M_1-2	Chr01	19	19	
M_17-2	Chr17	15	15	
M_12-1	Chr12	17	17/18	
M_5-5	Chr05	17	17	
M_X-1	X	27	27	
M_13-1	Chr13	17	17	
Human D4/D8	human	-	-	-

Microsynth AG
Schützenstrasse 15 · CH-9436 Balgach · Phone +41 71 722 83 33 · info@microsynth.ch · www.microsynth.com

4.2. Electropherogram

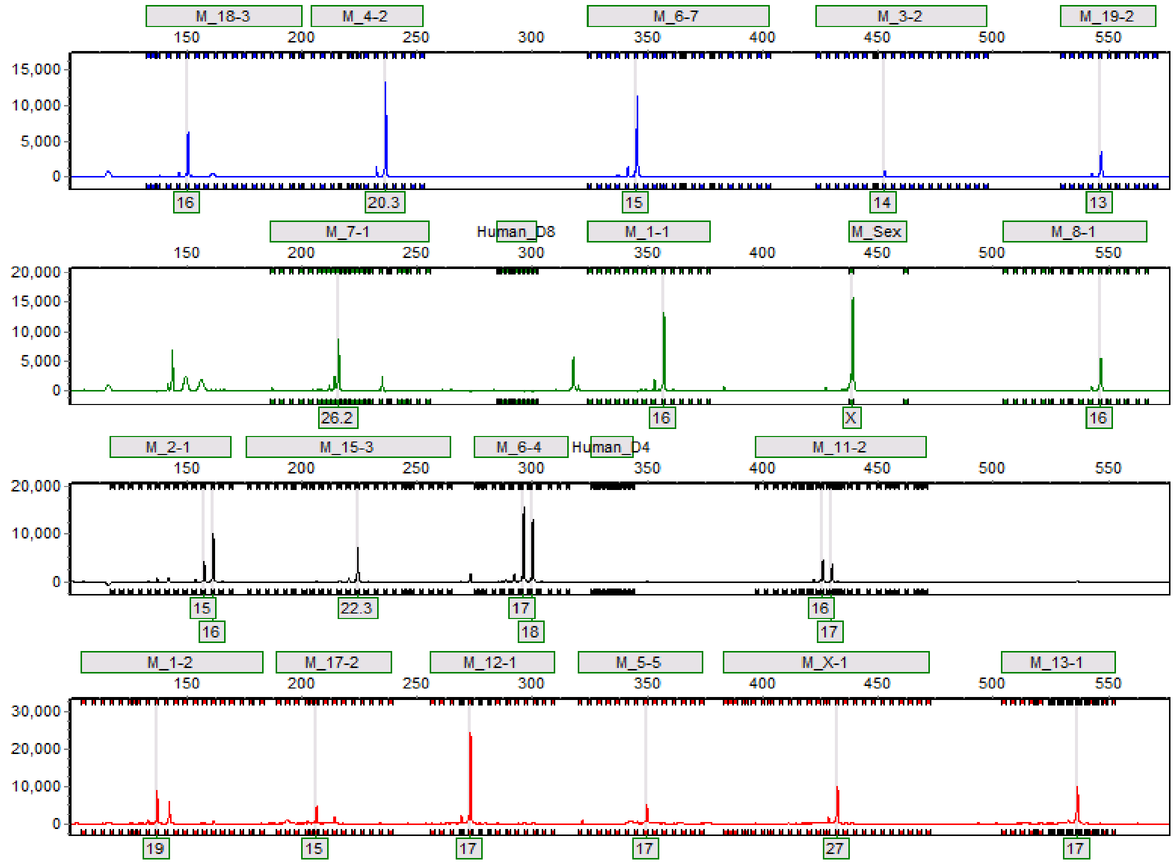

5. Conclusion

According to our analysis of the submitted sample there is no detectable contamination with human origin.

The analyzed data of the submitted sample match 86.1 % to the DNA profile of the cell line AT-3 (Cellosaurus, RRID:CVCL_VR89).

6. Customer Comment

No specific customer comments were provided for this test item

7. Reference

Almeida JL, Dakic A, Kindig K, Kone M, Letham DLD, Langdon S, Peat R, Holding-Pillai J, Hall EM, Ladd M, Shaffer MD, Berg H, Li J, Wigger G, Lund S, Steffen CR, Fransway BB, Geraghty B, Natoli M, Bauer B, Gollin SM, Lewis DW, Reid Y. Interlaboratory study to validate a STR profiling method for intraspecies identification of mouse cell lines. PLoS One. 2019 Jun 20;14(6):e0218412. doi: 10.1371/journal.pone.0218412. PMID: 31220119; PMCID: PMC6586308.

8. Glossary

Short Tandem Repeats (STRs)

Short tandem repeats (STRs) consist of a DNA motif of 2-13 bases that are repeated up to several hundred times. The number of repeats in a STR is highly variable among individuals, resulting in fragment length differences if amplified using PCR. These differences in fragment lengths at different loci are used for profiling the cell lines.

Stutter Peaks

Stutter peaks are small peaks which occur immediately before or after the true peak. Stutter peaks are commonly caused by a slippage of the polymerase during the PCR amplification.

Detection of Cell Line Mixtures

A mixture of two or more mouse cell lines is difficult to detect due to the nature of the matter. To detect interspecies contamination the STR profiling assay contains two human STR markers. If there is evidence of a clear intra- or interspecies contamination, we will comment the finding in the conclusion of the report.

Peak height ratio

Peak height ratio <25% (to the highest peak within a STR) is mentioned in the summary table (comments). Peak height ratios <25% need not necessarily have an effect on the behaviour or characteristics of the cell line. A small peak height may be due to reduced amplification efficiency for example resulting from a mutation in the primer site. The reason for the difference in peak heights observed, however, would need some in depth analysis of the test item.

9. General Comment

The results refer only to the portion of the sample Microsynth has analyzed. The analysis results might not be assigned unconditionally to the whole sample. Microsynth shall not in any event be liable for incidental, consequential or special damages in relation to carried out analysis and corresponding results.

This report is the confidential property of the client addressed. The report may only be reproduced in full. Publication of extracts from this report is not permitted without written approval from Microsynth.

10. Compliance and Quality Assurance Statement

All aspects of this study were in accordance with ISO 9001:2015 standards. All the applied equipment is qualified and calibrated. The applied methods are validated.
